# Acculturation, resilience, and healthcare trust in migrant populations: an equity perspective

**DOI:** 10.1186/s13690-026-01946-8

**Published:** 2026-06-04

**Authors:** S. Grüll, J. Huebner, Gianluca Ciarlo

**Affiliations:** 1 Klinik für Innere Medizin II, Universitätsklinikum Jena, Am Klinikum 1, Jena, 07747 Germany; 2 Medizinische Klinik I, Universitätsklinikum Frankfurt, Theodor-Stern-Kai 7, Frankfurt am Main, 60596 Germany; 3 Medical Clinic 1 Gastroenterology, Hepatology, Pneumology, Allergology, Nutritional Medicine, Endocrinology, University Hospital Frankfurt, Theodor-Stern-Kai 7, Frankfurt am Main, 60590 Germany

**Keywords:** Physician-Patient Relations, Resilience, Psychological, Cultural Competency, Trust, Acculturation, Emigrants and Immigrants

## Abstract

**Background:**

Trust in the doctor-patient relationship is crucial for treatment outcomes. Acculturation influences health literacy, attitudes toward medical authority, and communication preferences, while resilience may affect how trust develops. This study examines how acculturation and resilience interact to shape trust in physicians, informing culturally sensitive, patient-centered care.

**Methods:**

Between October 2023 and January 2024, 375 patients were surveyed in a monocentric primary care study using an anonymous questionnaire on acculturation, resilience, trust in medical care and health-related perceptions.

**Results:**

Among migrants, 47.9% were born in Germany; 80.8% had lived in Germany > 20 years. Acculturation was predominantly German, higher in women (M = 3.77 vs. 3.46) and younger respondents (M = 3.38 in 18–39 years, M = 3.42 in 40–59 years, and M = 2.75 in ≥ 60 years).Trust in physicians was high overall, but lower in migrants (M = 2.94 vs. 3.03) and younger adults (M = 2.90 in participants aged 20–39 years vs. M = 3.25 in those aged ≥ 60 years). Resilience was almost even across gender (M = 3.63–3.64), slightly lower in migrants (M = 3.48 vs. 3.66), and increased with age (M = 3.55 in 20–39 years, M = 3.60 in 40–59 years, and M = 3.78 in ≥ 60 years). Logistic regression showed no predictive link between resilience and migration status (*p* = 0.913).

**Conclusion:**

Acculturation proved to be a key factor influencing trust in doctors and mental stability, with women and younger patients showing higher levels of integration. Resilience also acted as a protective factor against acculturation stress and increased with age. Differences in trust indicate unequal access to culturally responsive care, highlighting the importance of linguistic integration and patient-centered communication.

**Trial registration:**

Not applicable, as this study was an observational, crosssectional survey without any interventional component and therefore did not meet criteria for trial registration.

**Table:** 

Text box 1. Contributions to the literature	
• This study conceptualizes trust in physicians as a population-level indicator of health system responsiveness, extending its relevance beyond individual clinical encounters. • It provides quantitative evidence on how acculturation shapes trust in primary care within a diverse, long-settled migrant population in a high-income health system. • The findings support a public health perspective linking migration, social integration, and communication to equity in access to patient-centered care. • By distinguishing acculturation from individual resilience, the study highlights why system-level approaches are needed to address trust-related health inequalities.	

## Background

The doctor-patient relationship and patients’ trust in medical expertise and therapeutic measures are considered key determinants of successful treatment. It is crucial that treating physicians are able to adequately perceive their patients’ needs and communicate them clearly [1]. According to scientific research, the doctor-patient relationship is shaped by elements such as communication, trust, participation, medical developments, social values, and established relationship models [2, 3].

Health literacy is an essential part of this interaction. It influences the extent to which patients can understand, evaluate, and incorporate medical information into their decisions [4]. Higher health literacy is associated with more constructive communication, greater involvement in treatment processes, and greater trust in medical care [5].

In vulnerable population groups, especially among migrants, trust in the doctor-patient relationship is particularly important. Language barriers, different experiences with national health systems, negative previous experiences, and uncertainties regarding residence status can hinder the development of trust and make it difficult to access professional care [6, 7]. Previous research has shown that migrants often report lower levels of trust in healthcare systems, which has been linked to communication barriers, perceived discrimination, and differing cultural expectations regarding medical care. These patterns have been observed across various countries and healthcare settings, underlining the international relevance of trust as a determinant of healthcare utilization and outcomes [6–8]. While these studies consistently highlight the relevance of trust in healthcare, findings remain heterogeneous across settings, and the underlying mechanisms linking migration-related factors to trust are not yet fully understood.

Acculturation, defined as adaptation to a new cultural environment while retaining elements of the culture of origin, influences health literacy, communication, and expectations toward medical care [9, 10]. Higher levels of acculturation have been associated with improved communication, increased health literacy, and greater trust in healthcare providers, suggesting that adaptation processes may play a key role in reducing disparities in healthcare experiences [9–11]. In this context, a lack of or impaired trust can lead to delayed use of medical services, reduced adherence to therapy, and increased use of informal or non-professional health resources [12]. At the same time, culturally determined differences in health literacy not only shape trust in medical care, but also resilience to health challenges [13]. Resilience, understood as the ability to maintain mental stability and capacity to act despite stressful circumstances, is influenced both culturally and by health literacy and, in turn, affects trust and the use of healthcare services [14, 15]. This reveals a close interrelationship between acculturation, health literacy, and trust, which significantly determines the quality and sustainability of the doctor-patient relationship.

Existing research has primarily focused on structural barriers and health literacy in migrant populations, while the role of psychosocial factors such as resilience in shaping trust in healthcare remains less well explored. In particular, the interaction between acculturation processes and individual resilience in influencing trust in physicians has received limited attention.

Taken together, existing research suggests that both structural and individual factors contribute to trust in healthcare, yet these dimensions are rarely examined in an integrated framework.

The conceptual framework of this study is based on the assumption that both structural and individual factors shape trust in healthcare. Acculturation is understood as a key process influencing patients’ ability to navigate the healthcare system and engage in communication, while resilience reflects individual coping resources in dealing with health-related challenges. These dimensions are assumed to interact in shaping trust in physicians, which is conceptualized as an indicator of healthcare system responsiveness and equity.

Against this background, this study examines how acculturation and resilience interact to shape trust in physicians, conceptualized as a marker of equitable access to patient-centered and culturally responsive care. The aim is to identify factors that are important for culturally sensitive care and patient-centered communication. Specifically, the study investigates the association between acculturation and trust in physicians, the relationship between resilience and trust, and the influence of sociodemographic factors such as age, gender, and migration background on these variables. In addition, it explores the combined contribution of acculturation and resilience in explaining variations in trust in physicians.

While previous research has examined structural barriers and health literacy in migrant populations, less attention has been paid to trust in physicians as an indicator of healthcare system responsiveness and equity, particularly in relation to acculturation processes. Furthermore, the interaction between acculturation and individual resilience remains insufficiently understood.

This study contributes to the literature by integrating acculturation and resilience as complementary dimensions to explain trust in physicians within a primary care setting. By conceptualizing trust as a marker of equitable access to patient-centered and culturally responsive care, the study provides a novel perspective linking individual adaptation processes with system-level responsiveness, and examines how these factors jointly influence trust across different sociodemographic groups.

## Methods

### Study population

This monocentric, prospective cross-sectional study was conducted in an outpatient primary care practice in southern Germany, providing care for approximately 4,000 patients annually, including a heterogeneous migrant population.

Eligible participants were adult patients (≥ 18 years) attending the practice during the study period who were able to provide informed consent and complete the questionnaire. Patients with insufficient language proficiency or cognitive impairment were excluded.

A consecutive sampling approach was applied, including all eligible patients during the recruitment period.

Between October 2023 and January 2024, 375 patients were enrolled using a standardized, anonymized questionnaire assessing acculturation, resilience, trust in healthcare, and health-related perceptions.

No formal a priori sample size calculation was performed, as the study followed an exploratory design. The sample size was determined by the number of eligible patients during the recruitment period. Although some eligible patients declined participation, the exact number of non-participants was not systematically recorded; therefore, a precise response rate cannot be reported.

Participants received written study information and provided informed consent prior to inclusion. Recruitment was conducted on site by trained personnel to ensure standardized administration of the study instruments. The study was conducted and reported in accordance with STROBE guidelines.

### Inclusion and exclusion criteria

Participants were included if they were capable of providing informed consent and possessed adequate language skills to complete the questionnaire either independently or with interpreter assistance. Individuals who did not meet these requirements were excluded from the study.

### Endpoints

The present study investigates the complex and dynamic interactions between acculturation, resilience, and trust in healthcare among migrants. The primary endpoint of this cross-sectional survey was to gain deeper insights into the factors that influence migrants’ trust in medical professionals and physicians. This knowledge is particularly relevant in the context of increasing migration and the growing importance of intercultural communication.

Secondary endpoints include examining how acculturation affects trust, the potential moderating role of individual resilience, and the interplay of these psychosocial factors in shaping healthcare utilization, with the ultimate goal of informing culturally sensitive and patient-centered medical practice.

### Questionnaire

A standardized questionnaire was developed to systematically assess the interplay between complementary and alternative medicine (CAM) use, resilience, acculturation, and trust in physicians and the healthcare system among patients with and without a migration background. CAM use was included as it may reflect culturally shaped health beliefs and trust in conventional healthcare systems.

The instrument was informed by a review of previously validated measures. Acculturation was measured using the Short Acculturation Scale for Hispanics (SASH), a validated 12-item instrument assessing language use, media language preferences, and ethnic social preferences [16]. Resilience was assessed using selected items from the Connor–Davidson Resilience Scale (CD-RISC) [17]. Trust in physicians and the healthcare system was measured using a set of Likert-scale items reflecting patients’ confidence in medical decision-making, perceived reliability, and overall trust in healthcare providers. Prior to full implementation, the questionnaire was pilot-tested with 20 patients to ensure clarity, feasibility, and content appropriateness.

The questionnaire comprised five interrelated domains: (1) sociodemographic characteristics and health status, (2) health-related attitudes and self-management, (3) trust in physicians and satisfaction with healthcare, (4) spiritual beliefs and practices, and (5) resilience-related capacities. This multidimensional approach enables a nuanced profiling of psychosocial and cultural factors influencing healthcare engagement in a primary care population with a high proportion of migrants.

#### Sociodemographic and health characteristics

Participants reported age, gender, country of birth, duration of stay in Germany, place of residence, religion, educational attainment, and current or past illnesses. Standardized closed-response formats with optional free-text fields allowed for structured data collection while capturing individual nuances. For participants with a migration background, additional variables were collected, including self-rated German language proficiency, health status, and language use across contexts such as reading, speaking, thinking, media consumption, at home, and in social situations. Social preferences concerning the cultural background of friends, social contacts, and children’s friendships were also documented.

#### Acculturation

Acculturation was assessed using the 12-item Short Acculturation Scale for Hispanics (SASH), a self-administered instrument measuring three dimensions: language use in different contexts (e.g., at home, work, and with friends), media language preferences, and ethnic social preferences. Although originally developed for Hispanic populations, the scale has been widely applied as a proxy measure of acculturation in diverse migrant groups, particularly with regard to language use and social integration. Items are rated on a 5-point Likert scale ranging from 1 (exclusive use of the native language) to 5 (exclusive use of German). A mean score was calculated across all items, with higher values indicating a stronger orientation toward German language use and social integration.

#### Health-related attitudes and self-management

Health-related attitudes and self-management were assessed using a set of items developed for this study, covering personal responsibility for health, perceived ability to prevent or manage illness, knowledge of medications and treatment options, confidence in implementing lifestyle changes, and engagement in health-promoting behaviors. While validated instruments for self-efficacy, such as the General Self-Efficacy Scale (SWE), are available, we chose a tailored approach to capture domain-specific aspects of health-related self-management relevant to the study context. The questionnaire was informed by previously validated measures and pilot-tested in a sample of 20 patients to ensure clarity and content validity. Items were rated on a Likert scale, and a mean score was calculated across all items, with higher values indicating greater perceived self-efficacy and engagement in health-related self-management. Internal consistency of the scale was good (Cronbach’s α = 0.85), with all items showing acceptable item–total correlations (> 0.39).

#### Trust in physicians and satisfaction with healthcare

Trust in physicians and satisfaction with healthcare were assessed using a set of items capturing key dimensions such as confidence in physicians’ decisions, perceived thoroughness and reliability, communication quality, perceived attention, and patient-centeredness. While established instruments for measuring trust and patient satisfaction exist, we chose a tailored approach to capture aspects most relevant to the specific study context and to allow integration with the other constructs assessed in this survey. The items were informed by previously validated measures. Internal consistency was good for the trust scale (Cronbach’s α = 0.82) and excellent for the satisfaction scale (Cronbach’s α = 0.94). Responses were recorded on a seven-point Likert scale ranging from “strongly disagree” to “strongly agree.” For both constructs, mean scores were calculated, with higher values indicating greater trust in physicians and higher satisfaction with healthcare, respectively. Total satisfaction scores were categorized as low (10–24 points), moderate (25–39 points), high (40–54 points), and very high (55–70 points).

#### Spiritual beliefs and practices

Items assessed the importance of prayer and personal reflection, perception of divine presence, and religious coping as sources of comfort and guidance. Responses were rated on four-point Likert scales from “does not apply” to “fully applies,” providing insights into existential and spiritual resources.

#### Resilience

Resilience-related capacities were evaluated via self-assessment of adaptive coping, stress management, persistence under adversity, flexibility, and emotional stability. Items were scored on five-point Likert scales from “not true at all” to “always true,” reflecting participants’ perceived ability to recover from stress and maintain psychological well-being. In addition, resilience was measured using the Connor-Davidson Resilience Scale (CD-RISC) [17], originally developed with 25 items scored on a 5-point scale (0–4), where higher scores reflect greater resilience. For this study, 8 items from the CD-RISC were selected. A mean score was calculated across the selected items, with higher values indicating greater resilience.

#### Complementary and alternative medicine (CAM) use and media trust

CAM engagement was measured using the German version of the International Complementary and Alternative Medicine Questionnaire (I-CAM-G) [18], employing dichotomous items (yes/no) for practices such as vitamin and micronutrient supplementation, herbal remedies, mistletoe therapy, acupuncture, yoga, and relaxation techniques. Participants could report additional practices via open-ended responses. Trust in information sources, including traditional media and social media, was assessed on a seven-point scale from “no trust at all” to “very high trust.”

This multifaceted tool enabled the investigation of the interrelationships between the use of complementary and alternative medicine practices, resilience, acculturation, and trust in physicians and the healthcare system, as well as their influence on health engagement and patient preferences within a culturally diverse primary care population.

### Statistical analysis

Statistical analyses were performed using IBM SPSS Statistics (version 29). Descriptive statistics—including frequencies, percentages, means, standard deviations, and ranges—were calculated to characterize the study population and summarize important variables such as acculturation, resilience, and trust in the healthcare system. Group differences, for example between participants with different levels of acculturation, were assessed using t-tests for independent samples for continuous variables and chi-square tests for categorical variables.

Multivariate linear regression analyses were performed to examine the relationships between acculturation, resilience, and trust in the healthcare system. Model assumptions, overall significance, regression coefficients, and 95% confidence intervals were evaluated to determine the strength and robustness of the observed relationships. All analyses were interpreted in the context of exploratory research, taking into account the cross-sectional design and observational nature of the study.

Potential sources of bias were considered during the analysis. Due to the lack of systematically recorded data on non-participants, a formal analysis of non-response bias was not feasible. Similarly, no distinction between early and late responders was performed.

As all data were collected using self-reported questionnaires, the possibility of common method bias cannot be excluded. However, validated instruments were used where available, and the questionnaire design aimed to minimize systematic response patterns.

Missing values were treated as system-missing. Descriptive statistics were calculated using all available data, while inferential analyses were based on complete cases (listwise deletion). No imputation procedures were performed.

### Ethics approval and consent to participate

Ethical approval for this study was obtained from the Ethics Committee of the University Hospital Jena and from the Ethics Committee of the State Medical Association of Baden-Württemberg (approval number 2023-3129-Bef, approved on October 16, 2023). The study was conducted in accordance with the ethical principles outlined in the Declaration of Helsinki (2013).

The study did not involve any experimental interventions, clinical trials, or animal experiments. Data were collected using an anonymous questionnaire. All participants were informed about the purpose of the study, assured of confidentiality, and provided written informed consent prior to participation. Participation was voluntary, and respondents could withdraw at any time without any consequences.

## Results

### Demographic data

Overall, between October 2023 and January 2024, 375 patients were included. Participants’ ages ranged from 18 to 88 years, with a mean of 50.3 years. The sample comprised 54.4% females, 45.1% males, and 0.3% non-binary/diverse individuals. Demographic data are summarized in Table 1.

**Table 1:** Demographic data

Category	Variable	*N* [= 375] (%) / value
Age	Mean (years) ± SD	50.30 ± 16.16
	Minimum (years)	18
	Maximum (years)	88
Gender	Female	204 (54.5)
	Male	169 (45.2)
	Diverse	1 (0.3)
	No answer	1
Migration background	Migrants	73 (19.5)
	Natives	302 (80.5)
Marital status	Single	63 (16.9)
	Married	233 (62.5)
	In a partnership / cohabiting	38 (10.2)
	Divorced	30 (8.0)
	Widowed	9 (2.4)
	No answer	2
Residence	Metropolis	14 (3.8)
	Rural area	353 (96.2)
	No answer	8
Religion	Christian	265 (71.2)
	Muslim	41 (11.0)
	Atheist / no religion	62 (16.7)
	Other	4 (1.1)
	No answer	3
Educational background	No school diploma	3 (0.8)
	Lower secondary school (Hauptschule)	141 (39.0)
	Intermediate secondary school (Realschule)	129 (35.6)
	University entrance qualification (Abitur) / applied sciences (Fachhochschulreife)	46 (12.7)
	University degree	43 (11.9)
	No answer	13
Number of children	None	104 (27.8)
	One	64 (17.1)
	Two	134 (35.8)
	More than two	72 (19.3)
	No answer	1
Chronic disease	None	180 (49.6)
	≥ 1	183 (50.4)
	No answer	12

*SD* Standard deviation, *n* number,

^1^chronical disease= Asthma, cardiac arrhythmia, and diabetes mellitus

### Participants with a migration background

Table 2 summarizes the demographic characteristics of participants with a migration background. Overall, 19.5% of respondents reported a migration background. Among these, 47.9% were born in Germany, 21.1% in Turkey, and 8.2% in Poland; the remaining participants originated from 12 other countries, totaling 15 nationalities. Most reported good or excellent German proficiency and health, and 80.8% had lived in Germany for more than 20 years.

**Table 2:** Demographic overview of patients with a migration background

Category	Variable	*N* [= 73] (%)
Country of birth	Germany	34 (47.9)
	Turkey	15 (21.1)
	Poland	6 (8.5)
	Hungary / Lithuania / Pakistan / Romania¹	8 (11.3)
	Bosnia-Herzegovina / Brazil / Croatia / Italy / Kazakhstan / Kyrgyzstan / Serbia / USA²	8 (11.3)
	No answer	2
Knowledge of German	Excellent	24 (33.3)
	Very good	16 (22.2)
	Good	19 (26.4)
	Sufficient	10 (13.9)
	Poor	3 (4.2)
	No answer	1
General state of health	Excellent	3 (4.2)
	Very good	10 (14.1)
	Good	38 (53.5)
	Sufficient	16 (23.0)
	Poor	4 (5.6)
	No answer	2
Years residing in Germany	1–5	1 (1.4)
	5–10	4 (5.5)
	10–20	9 (12.3)
	≥ 20	59 (80.8)

^1^ two patients per country

^2^one patient per country. Percentage may not sum to 100% due to rounding

### Acculturation among patients with migration background

The analyses of the acculturation items reveal that respondents’ linguistic and social orientation is predominantly German overall (Table 3). In terms of language, mean values range from M = 2.84 for language used in childhood to M = 3.92 for radio language, indicating a clear shift toward predominantly German-language everyday communication. Linguistic adaptation is particularly evident in communication with friends (M = 3.63), with women showing higher values than men (M = 3.77 vs. 3.46). A similar pattern is observed across age groups for general language use (M = 3.38 among 18–39-year-olds; M = 3.42 for 40–59-year-olds; M = 2.75 for those ≥ 60 years), indicating that younger respondents are more strongly oriented toward German, while older groups show lower levels of acculturation.

**Table 3:** Acculturation patterns: mean scores of German language use across gender and age

Acculturation – language items						
Variable	Women	Men	18–39 years	40–59 years	≥ 60 years	Overall
General language	3.31	3.22	3.38	3.42	2.75	3.29
Childhood language	2.83	2.78	2.96	2.91	2.33	2.84
Language at home	3.37	3.42	3.60	3.45	3.00	3.42
Thinking language	3.26	3.49	3.62	3.58	2.50	3.40
Language with friends	3.77	3.46	3.69	3.85	3.00	3.63
TV language	3.71	3.54	3.73	3.88	2.92	3.64
Radio language	3.91	3.89	4.12	4.09	3.17	3.92
Media preference	3.89	3.57	4.00	3.91	2.83	3.74

Acculturation – social items						
Variable	Women	Men	18–39 years	40–59 years	≥ 60 years	Overall
Close friends	3.60	3.47	3.73	3.55	3.18	3.56
Visiting contacts	3.63	3.58	3.69	3.70	3.36	3.63
Children’s friendships	3.91	3.44	3.79	3.62	3.45	3.63
Social gatherings	3.76	3.22	3.25	3.59	3.80	3.46

The social acculturation items (close friends, visiting contacts, children’s friendships, social gatherings) range between M = 3.46 and M = 3.63 overall. These values indicate that participants maintain social contacts both within their culture of origin and with the German majority society. Differences are also observed by gender and age: women show values between M = 3.60 (close friends) and M = 3.76 (social gatherings), while men range between M = 3.22 and M = 3.58. Across age groups, participants aged 18–39 years demonstrate consistently higher values (e.g., close friends M = 3.73), while those aged 40–59 years show similarly stable values (e.g., social gatherings M = 3.59). Participants aged ≥ 60 years tend to have lower values (e.g., close friends M = 3.18).

### Trust in physicians across sociodemographic groups

Overall, trust in physicians was high. Native Germans reported slightly higher trust levels than participants with a migration background. Differences were also observed across age groups, with younger participants showing lower levels of trust and increasing values with age. A similar pattern was observed for gender, with men reporting slightly higher trust than women (Table 4).

**Table 4:** Trust in physicians by sociodemographic characteristics

Group	Mean	SD	*N*
Migration status			
Native Germans	3.03	0.48	289
Migrants	2.94	0.50	66
Age groups [in years]			
18–39	2.90	0.49	99
40–59	2.97	0.48	150
60–75	3.15	0.41	91
76–100	3.25	0.69	15
Gender			
Men	3.05	0.42	160
Women	2.98	0.53	193

*SD* Standard deviation, *n* number

### Demographic patterns in self-reported resilience

**Fig. 1:**
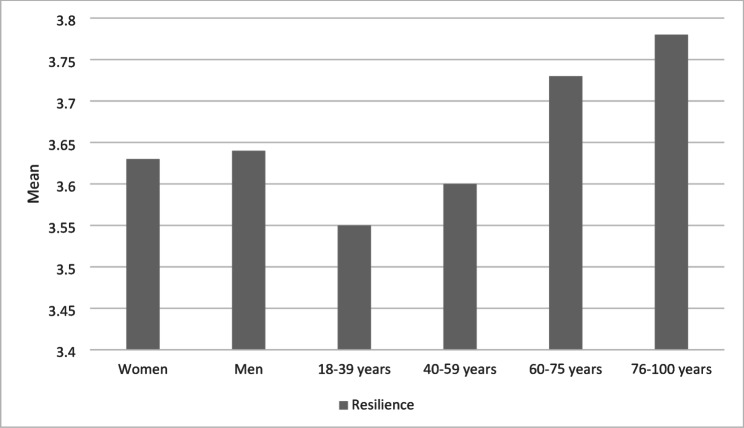
Descriptive overview of resilience levels

The overall sample showed a stable level of resilience with only minor sociodemographic differences (Fig. 1). Gender proved to be insignificant (women: M = 3.63; men: M = 3.64). Native participants reported slightly higher scores (M = 3.66) than people with a migration background (M = 3.48), with the latter showing greater variability. The most pronounced difference was an age-related increase: from the lowest scores among 18–39-year-olds (M = 3.55) to average scores among 40–59-year-olds (M = 3.60) to the highest scores among 60–75-year-olds (M = 3.73) and 76–100-year-olds (M = 3.78; small sample size).

A binary logistic regression to predict migration status based on the mean resilience score did not reveal any significant correlation (B = − 0.012; SE = 0.107; Wald = 0.012; *p* = 0.913; Exp(B) = 0.988). This underscores that although differences in resilience levels between native and migrant participants are descriptively visible, they cannot be considered statistically predictive.

## Discussion

The findings of this study address the research questions by demonstrating that acculturation and resilience are differentially associated with trust in physicians and are influenced by sociodemographic factors. A pattern of German-dominated bilingualism and moderate to high levels of social integration was observed, with higher levels of acculturation among women and younger respondents, while older participants showed lower levels of adaptation. Differences in trust between migrant and native-born patients should not be interpreted solely as individual attitudes but may reflect unequal conditions within healthcare interactions, including differences in access to linguistically and culturally responsive communication. Lower trust among migrants and younger patients may therefore indicate structural asymmetries in recognition, participation, and perceived responsiveness of the healthcare system. In this context, acculturation may facilitate navigation of medical encounters and reduce perceived power imbalances in the physician–patient relationship.

Native participants, older respondents, and men tended to report slightly higher levels of trust, whereas younger individuals and migrants showed somewhat lower levels. These differences were moderate, suggesting that trust is shaped by a combination of origin, age, and gender.

Our data suggest that acculturation is not a linear process, but is influenced by life stage and social roles. Social roles, such as partnership and employment, further modulate these relationships [19]. In particular, the acculturation strategies of “integration” and “assimilation” are associated with better quality of life scores and fewer depressive symptoms, while “separation” and “marginalization” are associated with poorer outcomes [11]. Comparative studies confirm this: Brand et al. [11] found that a higher degree of acculturation among Turkish migrants was associated with better Health-related Quality of Life (HRQoL). Further studies confirm that acculturation influences not only social participation but also mental health, with generational and gender-specific differences [19]. Promoting integration processes and taking individual circumstances into account are therefore key starting points for improving quality of life and mental health in migrant populations [11, 20].

Acculturation acts as a resource for mental stability by supporting both linguistic and social orientation [21, 22]. A good command of German facilitates participation in social life and access to support services, which has been associated with higher quality of life, lower levels of depression, and better mental health in previous studies. At the same time, social networks, friendships, and integration into local communities strengthen resilience and protect against depressive and post-traumatic symptoms [23, 24]. A balance between integration into the host society and preservation of one’s own cultural identity has been shown to be particularly beneficial for mental well-being [25]. Against this background, promoting linguistic and social integration, alongside structurally anchored patient-centered communication, is essential for reducing inequities and strengthening trust in healthcare systems.

Acculturation processes, especially language proficiency and social integration, are crucial for trust in physicians and patient satisfaction among migrants and younger patients. Good German language skills facilitate communication, improve understanding of medical information, and promote active participation in treatment decisions, while deficits lead to misunderstandings, dissatisfaction, and a greater need for interpreter support [8, 26]. Social integration, for example through stable networks and longer periods of residence, is associated with respectful treatment, higher trust, and a more positive perception of the responsiveness of the healthcare system [27, 28]; younger and more integrated patients also show more active participation in treatment [28, 29]. Overall, language and integration have a direct impact on subjective health and quality of life, making their promotion central to sustainably improving trust, patient satisfaction, and health satisfaction.

Resilience acts as a general protective factor for mental stability in patients with a migration background. However, the present findings suggest that resilience alone does not explain differences in trust or healthcare experiences [30]. Resilience appeared to vary primarily with age, while gender differences were minimal. Differences by migration status were less pronounced in terms of mean values but reflected greater variability within migrant groups. Resilient individuals show less psychological distress such as depression, anxiety disorders, or post-traumatic stress disorder, even when exposed to high levels of discrimination or post-migrant stressors [31]. In conjunction with acculturation, resilience supports the successful management of adaptation processes, promotes trust in doctors, and strengthens the ability to positively reevaluate and actively shape one’s own life. In this context, integration and social identity act as stabilizing resources [30, 32]. Resilience increases psychological resilience and reduces the effects of acculturation stress on well-being, without, however, moderating the direct effects of trauma or discrimination [33, 34]. With increasing age and life experience, resilience continues to improve, enabling better coping with migration-related challenges and greater trust in the healthcare system [30]. Overall, rather than compensating for structural or communicative inequities, resilience appears to operate within the constraints of existing healthcare conditions, underscoring that individual coping resources cannot substitute for system-level equity and responsiveness. These findings extend existing research by highlighting that acculturation and resilience should be considered jointly when examining trust in healthcare.

The results of this study and the existing literature show that acculturation has a central influence on trust in physicians and mental health. Interventions to promote linguistic and social integration could improve health-related quality of life and strengthen trust in the healthcare system. Future research should examine the interactions between acculturation, resilience, and trust more closely in order to develop evidence-based, culturally sensitive care strategies.

### Limitations and future directions

This study has several limitations. It was conducted in a single primary care practice, which may limit the generalizability of the findings to other healthcare settings. The single-center design may have introduced selection bias, as the patient population reflects the specific characteristics of this practice. Participation was voluntary, which may have led to underrepresentation of certain groups, particularly less integrated migrant patients and those with language barriers or concerns about the length of the questionnaire.

From a methodological perspective, the cross-sectional design precludes causal inferences regarding the relationships between acculturation, resilience, and trust in healthcare. In addition, the reliance on self-reported data introduces the potential for reporting bias and common method bias, which may have influenced the observed associations. Furthermore, the use of partially adapted and self-developed instruments, although guided by validated measures and supported by acceptable internal consistency, may limit comparability with other studies and affect construct validity.

From a theoretical perspective, acculturation and resilience are complex, multidimensional constructs that cannot be fully captured by standardized questionnaire measures alone. The operationalization used in this study focuses primarily on language use, social integration, and individual coping resources, and may therefore not fully reflect broader structural, cultural, and contextual influences on healthcare experiences.

In addition, the literature review was restricted to English- and German-language sources within a defined time frame, which may have excluded relevant publications in other languages or from earlier periods. Furthermore, most migrant participants had lived in Germany for more than two decades, indicating a relatively high level of integration and familiarity with the healthcare system. This may limit the applicability of the findings to more recently arrived or less integrated migrant populations.

Future research should address these limitations by including more diverse and less integrated migrant populations, as well as by applying longitudinal designs to better understand causal relationships between acculturation, resilience, and trust in healthcare. From a clinical perspective, the findings highlight the importance of culturally and linguistically sensitive communication strategies. Interventions such as improved language support and patient-centered communication approaches may help to strengthen trust in physicians, particularly among migrant and younger patient groups. At the system level, promoting culturally responsive care structures and integrating intercultural competence training into medical education may contribute to reducing healthcare inequities and improving patient experience.

## Conclusion

The present findings demonstrate that acculturation and resilience are important, yet distinct, factors shaping trust in physicians and mental well-being. Acculturation emerges as a key mechanism facilitating communication, participation, and navigation within the healthcare system, while resilience supports individual coping but does not fully compensate for structural and communicative barriers.

These results suggest that trust in healthcare is not solely determined by individual characteristics, but is shaped by the interaction between personal resources and system-level conditions. In this context, linguistic and social integration appear to be central determinants of equitable healthcare experiences.

Strengthening culturally and linguistically responsive care, alongside promoting patient-centered communication, may therefore represent essential steps toward improving trust in healthcare systems, particularly among migrant and younger populations. Future research should further investigate the interplay between acculturation, resilience, and trust using more diverse populations and longitudinal designs.

## Data Availability

No datasets were generated or analysed during the current study.
